# Genetic dissection of cold tolerance across developmental stages in sorghum reveals stage-specific haplotypes and potential trade-offs

**DOI:** 10.1007/s00122-026-05356-w

**Published:** 2026-09-04

**Authors:** Mohamed Mosalam, Hannah Robinson, Steffen Windpassinger, Rod J. Snowdon, Kai P. Voss-Fels

**Affiliations:** 1 Department of Plant Breeding, Hochschule Geisenheim University, Geisenheim, Germany; 2 Department of Plant Breeding, Justus Liebig University Giessen, Giessen, Germany

## Abstract

**Key message:**

Cold tolerance in sorghum is largely stage- and environment-specific yet partially shared genetic control allows improvement across developmental stages with mostly modest, trait-specific trade-offs.

**Abstract:**

Cold tolerance is a major constraint to sorghum production in temperate environments, and its improvement is therefore a key breeding objective. However, cold tolerance varies across developmental stages and environments, and selection based on stage-specific phenotypes may overlook shared genetic components. We analysed a diversity panel of 394 sorghum genotypes evaluated across 10 environments representing three developmental stages using multi-environment mixed models, genome-wide haplotype block analysis, and in silico haplotype stacking. Substantial genotype-by-environment interaction was detected, with the strength of GEI varying markedly across traits. Genetic correlations were high within developmental stages but lower and more variable across stages, indicating that the genetic architecture of cold tolerance-relevant traits is largely stage-dependent. Haplotype block analyses identified both stage- and environment-specific regions, as well as regions shared across stages and environments. In silico stacking of favourable haplotypes for early-stage cold tolerance resulted in nonlinear predicted gains, with strong initial responses at low stacking levels followed by diminishing returns. Correlated responses in yield-related traits were generally positive, whereas flowering time was consistently delayed, indicating a potential trait-specific trade-off in cold tolerance improvement. Sorghum cold tolerance can therefore not be treated as a single uniform trait but rather as a combination of stage-specific and shared genetic components, with implications for multi-stage breeding strategies.

**Supplementary Information:**

The online version contains supplementary material available at https://doi.org/10.1007/s00122-026-05356-w.

## Introduction

Sorghum (*Sorghum bicolor* [L.] Moench) is a C4 cereal crop that exhibits high photosynthetic efficiency and requires less water and fertiliser inputs than many other cereals (Martínez‐Goñi et al. 2022). It ranks as the fifth most important cereal globally and serves diverse end uses, including food, fodder, and bioenergy production (FAOSTAT 2024). Sorghum is also nutritionally valuable, being rich in protein and fibre, and naturally gluten-free (Tanwar et al. 2023). Domesticated in north-eastern Africa, sorghum is widely cultivated in warm, arid regions due to its resilience to drought and ability to thrive under conditions unsuitable for many other crops (Burgarella et al. 2021). However, sorghum has poor adaptation to low temperatures, which restricts its distribution and expansion into temperate and high-altitude regions (USDA 2012).

The impact of cold stress on sorghum varies markedly across developmental stages. During germination, temperatures below 8.7 °C can prevent or delay emergence (Fiedler et al. 2016; Kravcov et al. 2025), while frost during the 4–6 weeks after emergence can cause severe (often fatal) damage to juvenile seedlings. In the vegetative phase (from seedling establishment to flowering), temperatures below 14.5 °C decrease seedling vigour, slow development, and delay flowering, all of which ultimately reduce yield potential (Emendack et al. 2021; Casto et al. 2021). At the reproductive stage, chilling stress can disrupt panicle development, reduce pollen viability, and impair grain filling, resulting in substantial yield losses (Chakrabarty et al. 2021; Neitzert et al. 2025, 2026). These stage-specific responses have distinct physiological and genetic bases (Casto et al. 2021), so effective breeding for cold tolerance should evaluate performance across the entire life cycle rather than focusing solely on survival.

Moreover, cold stress during early stages can have carry-over effects on subsequent development, delaying flowering and maturity and ultimately reducing yield at harvest (Maulana and Tesso 2013; Emendack et al. 2021). This suggests that cold responses can be linked across developmental stages, rather than being confined to the period of exposure (Hernández et al. 2023). Therefore, improving cold tolerance throughout the life cycle would enable earlier sowing, more reliable establishment, and greater yield stability.

Accordingly, cold tolerance is typically inferred from proxy traits relevant to each developmental stage. For early-stage tolerance, frost survival percentage serves as a key indicator for germination under cold stress (Patanè et al. 2021). Seedling vigour is widely adopted as an indicator during the seedling stage because it reflects the capacity to maintain growth under chilling stress (Maulana and Tesso 2013; Upadhyaya et al. 2015). However, seedling vigour is often measured at a single time point, which may not fully capture variation in growth rate throughout the seedling stage (Agnew et al. 2024). At the reproductive stage, flowering time provides a measure of maturity (Maulana and Tesso 2013; Emendack et al. 2021) and reflects the interaction of the genotype with the post-germination temperature conditions. Ultimately, yield-related traits anchor breeding efforts by linking cold tolerance to harvest performance (Chakrabarty et al. 2021). Here, “cold tolerance” encompasses the ability of sorghum to germinate, sustain growth, and complete reproduction and grain filling under low-temperature conditions (Hernández et al. 2023).

Genotype-by-environment (G×E) interactions further complicate genetic improvement, as genotypes that perform well under cold stress may exhibit reduced performance under optimal or warm conditions (Hernández et al. 2023). For instance, selection for cold tolerance in Chinese landraces has been shown to reduce performance in favourable environments (Yang et al. 2025). Field trials across multiple stress environments reveal low-to-moderate correlations among trials (r = 0.1–0.4) and substantial genotype re-ranking (Marla et al. 2019), while concordance between semi-controlled and open-field performance is often poor (Knoll and Ejeta 2007). These findings indicate that selection in a single environment rarely yields consistent improvement, highlighting the necessity of multi-environment trials (METs) to achieve stable performance across varying conditions.

Consequently, improving cold tolerance in sorghum requires integrative analyses capable of quantifying genetic correlations among environments (Teng et al. 2024), distinguishing shared from stage-specific genetic control (Chen et al. 2023), and detecting pleiotropy among traits (El-Wahab et al. 2023). This information is essential to determine whether cold-response traits can be improved simultaneously or are limited by genetic trade-offs (Yadav et al. 2025). To address these challenges, analytical frameworks are required that jointly model genetic correlations across environments and developmental stages. Here, we apply a unified genomic best linear unbiased prediction (GBLUP) mixed-model framework to analyse cold-response traits across multi-environment trials (METs) and developmental stages. Within this framework, genotype-by-environment and genotype-by-time point interactions are modelled using flexible genetic (co)variance structures, allowing for heterogeneous genetic variances and estimation of genetic correlations across environments and developmental stages.

However, current approaches often rely on single-marker effects (Mosalam et al. 2025), which can underestimate the combined contribution of linked variants. In contrast, haplotype blocks aggregating neighbouring markers provide a more biologically meaningful representation of genomic segments inherited as units through breeding (Sivabharathi et al. 2024; Weber et al. 2023). This is particularly relevant for cold tolerance, where pleiotropic and stage-specific responses may arise from linked variants (Singh et al. 2025). Haplotype-based analyses can thus facilitate prioritisation of genomic regions with stable, stage-specific, or antagonistic effects across traits and environments, yielding more relevant targets for breeding decisions (Bhat et al. 2021). These considerations underscore the need for integrated analyses that link cold-response traits across developmental stages and environments to interpretable genomic regions. Therefore, the objectives of this study are to: i) characterise the genetic architecture of cold-response traits in sorghum across developmental stages and environments, ii) quantify genetic correlations and associated trade-offs among cold-response traits to assess the potential for simultaneous improvement, and iii) identify haplotype blocks associated with cold-response traits, including pleiotropic regions relevant to breeding.

### Experimental design and genetic material

A diversity panel of 394 *Sorghum bicolor* accessions was used to investigate the genetic architecture of cold tolerance. The panel consisted of recombinant inbred lines representing the five botanical races of sorghum (bicolor, caudatum, durra, guinea, and kafir) as well as inter-race crosses*.* These accessions originated from 24 countries and were previously characterised by Chakrabarty et al. (2021) and Kravcov et al. (2025). The panel also included conversion lines, temperate-adapted lines and German breeding lines representing grain, silage, and dual-purpose end uses. All lines were genotyped using DArTseq (Diversity Arrays Technology Pty Ltd, Canberra, Australia), yielding 26,286 SNP markers for downstream analyses. This panel has been previously characterised for multiple traits including juvenile frost tolerance, reproductive cold tolerance, plant height, flowering time and yield-related traits (Chakrabarty et al. 2021; Kravcov et al. 2025) and exhibits substantial genetic diversity for genetic analysis and breeding.

**Fig. 1:**
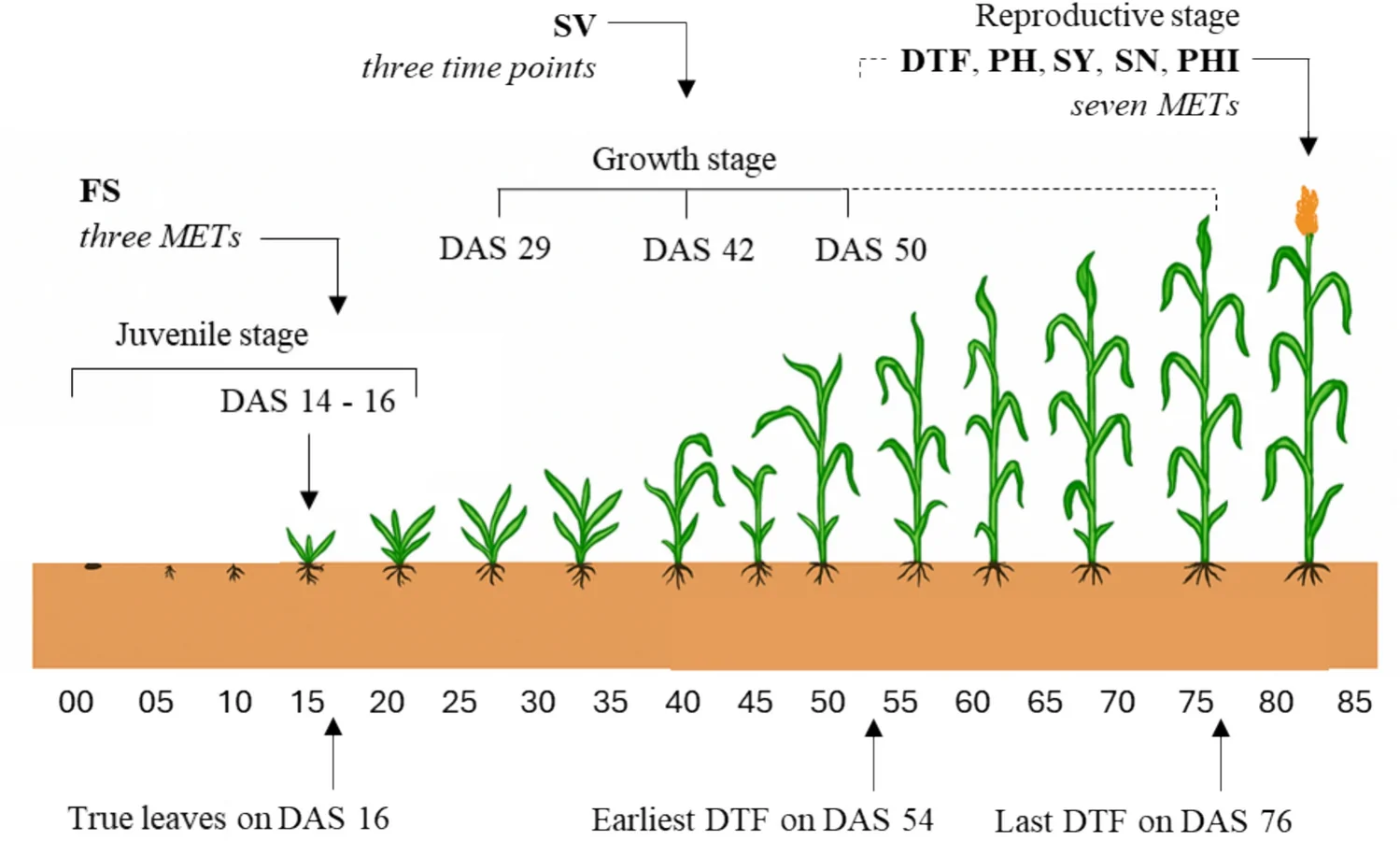
Developmental stages and phenotyping timeline. The horizontal numbers indicate days after sowing (DAS). Frost survival (FS) was scored during the juvenile stage (14–16 DAS) in three environments. Seedling vigour (SV) was recorded at 29, 42, and 50 DAS during the growth stage. Reproductive-stage traits: days to flowering (DTF), plant height (PH), seed yield (SY), seed number (SN), and panicle harvest index (PHI) were assessed across seven environments. METs indicate multi-environment trials

This study utilised phenotype data from two parallel experiments designed to evaluate cold tolerance across sorghum developmental stages (Fig. 1). The phenotypic data and detailed field trial descriptions were previously reported by Chakrabarty et al. (2021) and Kravcov et al. (2025). In total, seven traits were assessed across 10 environments (Table 1).

**Table 1:** Environmental conditions and experimental design of the ten trials across the three developmental stages

Stage	Env	Location	Country	Year	Coordinates	Altitude	Soil type	Mean temp °C	Mean max temp °C	Mean min temp °C	Precipitation (mm/growing season)
Reproductive stage	Asendorf (AS17)	North German Plain	Germany	2017	52°46 ‘N, 09°01 ‘E	49 m	Loamy sand	16.1	21.0	11.6	611
	Poel (PL17)	Baltic Sea Island	Germany	2017	53°99’N, 11°47’E	19 m	Loamy sand	16.7	20.2	13.1	301
	Rauischholzhausen (RH17)	Central German uplands	Germany	2017	50° 45′ 35″ N, 08° 53′ 04″ E	235 m	Clay	16.0	21.8	11.1	632
	San Juan del Río (SJR17)	Central Mexican Plateau	Mexico	2017	20°25 ‘N, 99°56 ‘W	1,920 m	Loam	23.0	31.4	14.7	326
	Texcoco (TEX17)	Valley of Mexico (Mexican High Plateau)	Mexico	2017	19°31 ‘N, 98°51 ‘W	2,250 m	Loam	16.4	24	8.7	360
	TEX18	Valley of Mexico (Mexican High Plateau)	Mexico	2018	19°31 ‘N, 98°51 ‘W	2,250 m	Loam	16.3	23.2	9.4	537
	Groß-Gerau (GG18)	Rhine–Main area	Germany	2018	49°55 ‘N, 08°29 ‘E	90 m	Sand	21.3	28.9	13.7	94 (+ 150 irrigation)
Growth stage	GG21	Rhine–Main area	Germany	2021	49°55 ‘N, 08°29 ‘E	90 m	Sand	9.7	17.0	2.8	NA
Juvenile stage	GG21	Rhine–Main area	Germany	2021	49°55 ‘N, 08°29 ‘E	90 m	Sand	9.7	17.0	2.8	NA
	JLU22	Giessen	Germany	2022	50.5805° N, 8.6771° E	90 m	potting substrate/ soil mix	2.4	19.2	-3	NA
	JLU23	Giessen	Germany	2023	50.5805° N, 8.6771° E	90 m	potting substrate/ soil mix	3.4	24.5	-3.3	NA

At the juvenile stage, frost survival (FS) was evaluated in three environments in Germany: an open-field trial in Gross-Gerau (49.92° N, 8.48° E) in 2021 (GG21) using an alpha-lattice design with two replications, and two semi-controlled trials at Justus Liebig University Giessen (50.5805° N, 8.6771° E) in 2022 (JLU22) and 2023 (JLU23), each implementing a randomised complete block design (RCBD) with three and four replications, respectively. Cold stress was induced in GG21 by sowing four weeks earlier than the recommended planting date, resulting in prolonged and variable freezing exposure. FS was assessed at 14–16 days after sowing (DAS) as the proportion of emerged seedlings surviving freezing (Kravcov et al. 2025). The GG21 trial was extended into the vegetative growth stage, where seedling vigour (SV) was recorded at 29, 42, and 50 DAS as the proportion of seedlings with healthy green leaves, no necrosis, and active growth.

Cold tolerance at the reproductive stage was evaluated in seven contrasting environments (Table 1), including four in Germany and three in Mexico. In Germany, trials were conducted in Gross-Gerau (49.92° N, 8.48° E) in 2018, and Rauischholzhausen (50° 45′ 35″ N, 08° 53′ 04″ E), Asendorf (52°46 ‘N, 09°01 ‘E), and Poel (53°99’N, 11°47’E) in 2017, corresponding to GG18, RH17, AS17, and PL17, respectively. In Mexico, trials were conducted at two high-altitude locations (approx. 2000-2200 m) in San Juan del Río (20°25 ‘N, 99°56 ‘W) in 2017, and Texcoco (Valley of Mexico (19°31 ‘N, 98°51 ‘W)) in 2017 and 2018, hereinafter referred to as SJR17, TEX17, and TEX18, respectively. In each environment, the population was divided into three adjacent blocks based on plant height to minimise shading, and within each block, an unreplicated RCBD was implemented. GG18 and SJR17 served as warm control conditions, while the remaining environments represented cold stress conditions. Grain yield (SY), seed number (SN), and panicle harvest index (PHI) were measured in all environments, while days to flowering (DTF) and plant height (PH) were only recorded in GG18, AS17, and PL17. DTF and PH were assessed on a plot basis when 50% of plants had flowered, with PH measured from the plant base to the panicle tip. Following harvest, five plants per genotype were randomly selected for yield assessments. SY was calculated as mean dry grain weight per plant, SN as mean seeds per plant, and PHI as the ratio of grain weight per panicle to total panicle weight before threshing (Chakrabarty et al. 2021), representing an efficient proxy for spikelet fertility (Krishnamurty et al. 2014).

### Genetic analysis using Restricted Maximum Likelihood (REML)

Phenotypic data were analysed using restricted maximum likelihood (REML) mixed models (Gilmour et al. 1995) implemented in ASReml-R v4.2 (VSNi, Hemel Hempstead, UK). For traits assessed in multi-environment trials (METs), linear mixed models estimated additive genetic, genotype-by-environment (G×E), and residual variance components (Smith and Cullis 2018; Lisle et al. 2021). For seedling vigour (SV), a repeated-measures mixed model was used to estimate additive genetic, genotype-by-time (G×T), and residual variance components (Apiolaza and Garrick 2001; Chaves et al. 2023). Genetic (co)variances across environments or time points were modelled with an unstructured (co)variance matrix (Butler et al. 2023).

### Population structure analysis and subpopulations

Population structure was visualised using principal component analysis (*PCA*) (Price et al. 2006). Subpopulations were subsequently identified using k-means clustering applied to the full PCA score matrix. The number of subpopulations (K) was chosen using the elbow method by plotting the within-subpopulation sum of squares (WSS) across increasing K and selecting the point at which the WSS curve began to plateau (Thorndike 1953), resulting in three subpopulations. The proportion of variance (PVE) was calculated as: $PVE=1-\frac{{WSS}_{K}}{TSS}$, where *TSS* is the total sum of squares. At K = 3, clustering explained 54.3% of the total variance; increasing K beyond 3 yielded progressively smaller gains in explained variance, supporting K = 3 as the elbow point. The results were used for subsequent analyses (Supplementary Figure S1).

### Model structures

The same mixed-model framework was used for both multi-environment (G×E) and repeated-measures (GxT) analyses. Model terms were incorporated sequentially guided by the experimental design and the biological relevance of each component and only those effects required to adequately account for the observed sources of variation were retained in the final models. Multi-environment models were applied to observations of genotype (j) in environment (i) and repeated-measures models to observations of genotype (j) at time point (t), as follows:

### Multi-environment model

$$y_{ijkl} = \mu + E_{i} + G_{j} + G \times E_{ij} + {\mathrm{Pop}}_{k} + {\text{ rep}}_{l\left( i \right)} + {\mathrm{spat}}_{l\left( i \right)} + \varepsilon_{ijkl}$$

### Repeated-measures model

$$y_{tjkl} = \mu + T_{t} + G_{j} + G \times T_{tj} + {\mathrm{Pop}}_{k} + {\mathrm{rep}}_{l\left( t \right)} + {\mathrm{spat}}_{l\left( t \right)} + \varepsilon_{tjkl}$$

where *y* is the phenotypic observation, *μ* is the overall mean, *E* and *T*​ are the fixed effects of environment and time point, *Pop*​ is the fixed covariate accounting for population structure by assigning genotypes to one of the three defined subpopulations, *G*​ is the random additive genetic effect of genotype, G×E and G×T are random genotype-by-environment and genotype-by-time interaction, *Rep* is the random replication, spat are the random spatial field design (rows, columns, block), and *ε*​ is the residual error.

Additive genetic effects were modelled using a genomic relationship matrix (G) constructed following VanRaden (2008):

$$G_{j} \sim N\left( {0,{\upsigma}_{g}^{2} G} \right),$$

where ​${\sigma}_{g}^{2}$ is the additive genetic variance.

Genotype-by-environment and genotype-by-time interactions were modelled as:

$$G \times E_{ij} \sim N\left( {0,\Sigma_{E} \otimes G} \right),$$

$$G \times T_{tj} \sim N\left( {0,\Sigma_{T} \otimes G} \right),$$

where ${\Sigma}_{E}$ and ${\Sigma}_{T}$ are unstructured covariance matrix allowing correlations among environments or time points, and ⊗ denotes the Kronecker product (Butler et al. 2023; Smith & Cullis 2018).

Residuals were modelled as:

$${\upvarepsilon } \sim N\left( {0,R} \right)$$

where R allows heterogeneous residual variances across environments or time points (De Faveri et al. 2022; Verbyla et al. 2021).

### Environmental clustering

Environmental clustering of the MET environments was performed using two complementary approaches based on different outputs from the MET models. First, genetic correlations among environments were derived from the estimated MET genetic (co)variance components:

$$r_{g} = \frac{{{\mathrm{Cov}}_{ij} }}{{\sqrt {Var_{i} \times Var_{j} } }},$$

where Cov_ij_​ is the genetic covariance between environments i and j, and Var_i_ and Var_j_​ are the corresponding genetic variances. These correlations were then transformed into a dissimilarity measure using genetic distance 1—r_g_, for hierarchical clustering. Thus, highly correlated environments were considered genetically similar and grouped together, whereas weakly correlated or negatively correlated environments were considered more distinct (Smith and Cullis 2018; Argaw et al. 2025).

Second, environment-specific G×E BLUP profiles were extracted from the MET model, arranged as an environment-by-genotype matrix, and summarised using PCA (Patterson et al. 2006). K-means clustering was then applied to the first two PCs to group environments with similar multi-variate G×E response patterns. The optimal number of clusters was selected as k = 2 using the elbow criterion.

### Estimation of heritability

Trait heritabilities were estimated for each environment cluster using Cullis’ heritability:

$${H}_{Cullis}^{2}=1-\frac{{\overline{v}}_{BLUP}}{2{\upsigma}_{g}^{2}}$$

where ${\overline{v}}_{BLUP}$ is the average pairwise prediction error variance of the difference between genotype BLUPs, and ${\sigma}_{g}^{2}$ is the genetic variance (Cullis et al. 2006).

### De-regression of GBLUPs

De-regressed predictions (DRPs) were computed from genotype BLUPs using genotype-specific reliabilities derived from PEV (Garrick et al. 2009). Because the dataset combined different experimental designs and unbalanced genotype replication across environments, BLUP reliabilities differed among genotypes. DRPs were therefore calculated for all traits to reduce reliability-dependent shrinkage and to generate adjusted genotype values for estimating genetic correlations between early- and late-stage traits. Reliabilities (*r*) were calculated as:

$${r}_{i}^{2}=1-\frac{{PEV}_{i}}{{\sigma}_{g}^{2}}$$

where PEV is the prediction error variance of each genotype BLUP, DRPs were then computed as:

$$DRP=\frac{BLUP}{r}$$

Reliabilities between 0.01 and 0.99 were retained to avoid extreme estimates (Butler et al. 2023). The resulting DRPs were then scaled before being used in the genetic correlation analysis.

#### Estimation of genetic correlations between early- and late-stage traits

Genotype data were converted into PLINK format and used to create a genomic relationship matrix (GRM) with GCTA v1.95.0 (Yang et al. 2010). Then, a multi-variate REML analysis was performed using MTG2 v2.08 (Lee and Van Der Werf 2016) to estimate the genetic variances and covariances for the traits.

#### Definition of genome-wide haplotype blocks based on LD decay

Linkage disequilibrium (LD) decay was used to define a genome-wide window size for haplotype block construction. LD (r^2^) was calculated from the genotype matrix following Hill and Weir (1988), as implemented by Remington et al. (2001), and a nonlinear least-squares fit of the Hill–Weir model was used to describe the decline of LD with increasing marker distance. Based on the overall LD decay pattern across chromosomes and subpopulations, a distance of 3 cM was considered a representative average extent of LD and was therefore used as a uniform window size for haplotype block construction by grouping adjacent markers within 3 cM windows in SelectionTools R (Weber et al. 2023). SNP effects were estimated by back-solving BLUPs obtained from both models using a mean-imputed centred genotype matrix, following:

$$\widehat{\upalpha }={\left({M}^{{\top}}M\right)}^{-1}{M}^{{\top}}\widehat{g}$$

where *M* is the genotypic matrix, with SNPs coded as 0, 1, and 2 according to the number of reference alleles carried by each genotype, and $\widehat{g}$ is the vector of genotype BLUPs (Liu et al. 2014). Haplotype block effects were calculated by aggregating SNP effects within each block to derive local genomic estimated breeding values (local GEBVs), and block-level variance (BlockVar) was calculated as the variance of haplotype effects within each block across individuals (Voss-Fels et al. 2019; Roy et al. 2025; Tong et al. 2025). Higher BlockVar values indicate greater differentiation among haplotypes within a block and were interpreted as indicative of a larger contribution of that block to genetic variation for the trait.

#### Assessment of haplotype effect stability between environmental clusters

Haplotype effect stability between E1 and E2 was estimated as the the Pearson correlation of haplotype effects within each block. Blocks were classified as stable (r > 0.80), moderately stable (0.50 ≤ r ≤ 0.80), or environment-specific (r < 0.50).

#### In silico haplotype stacking and trade-off analyses

Haplotype stacking and associated trade-offs were evaluated within a local haplotype effect framework. Since alternative haplotypes within a block can confer trait-specific effects, block effects varied among genotypes. For each block, the genotype exhibiting the most favourable block effect for the target trait was designated as the donor. The stacking analysis was performed on nine recipient genotypes, selected as the top three genotypes from each of the three PCA-defined clusters. Block stacking was simulated by sequentially replacing the block-specific effect of each recipient genotype with that of the donor, recalculating the predicted phenotype after each additional stacked block (Villiers et al. 2024). Trade-offs were assessed as responses in non-target traits by applying the same set of top-ranked blocks identified for the target trait and evaluating their corresponding effects on non-target traits.

#### Pleiotropy assessment

Haplotype blocks were ranked by their cross-haplotype variance for each trait from highest to lowest. The top 10% of blocks per trait, i.e. blocks exhibiting strongly contrasting haplotype effects, were retained. Subsequently, putatively pleiotropic (i.e. shared) blocks were defined as those appearing in the top-decile sets of at least two traits, following the variance-based framework of Tong et al. (2024).

## Results

### PCA defines subpopulations and supports environment grouping

**Fig. 2:**
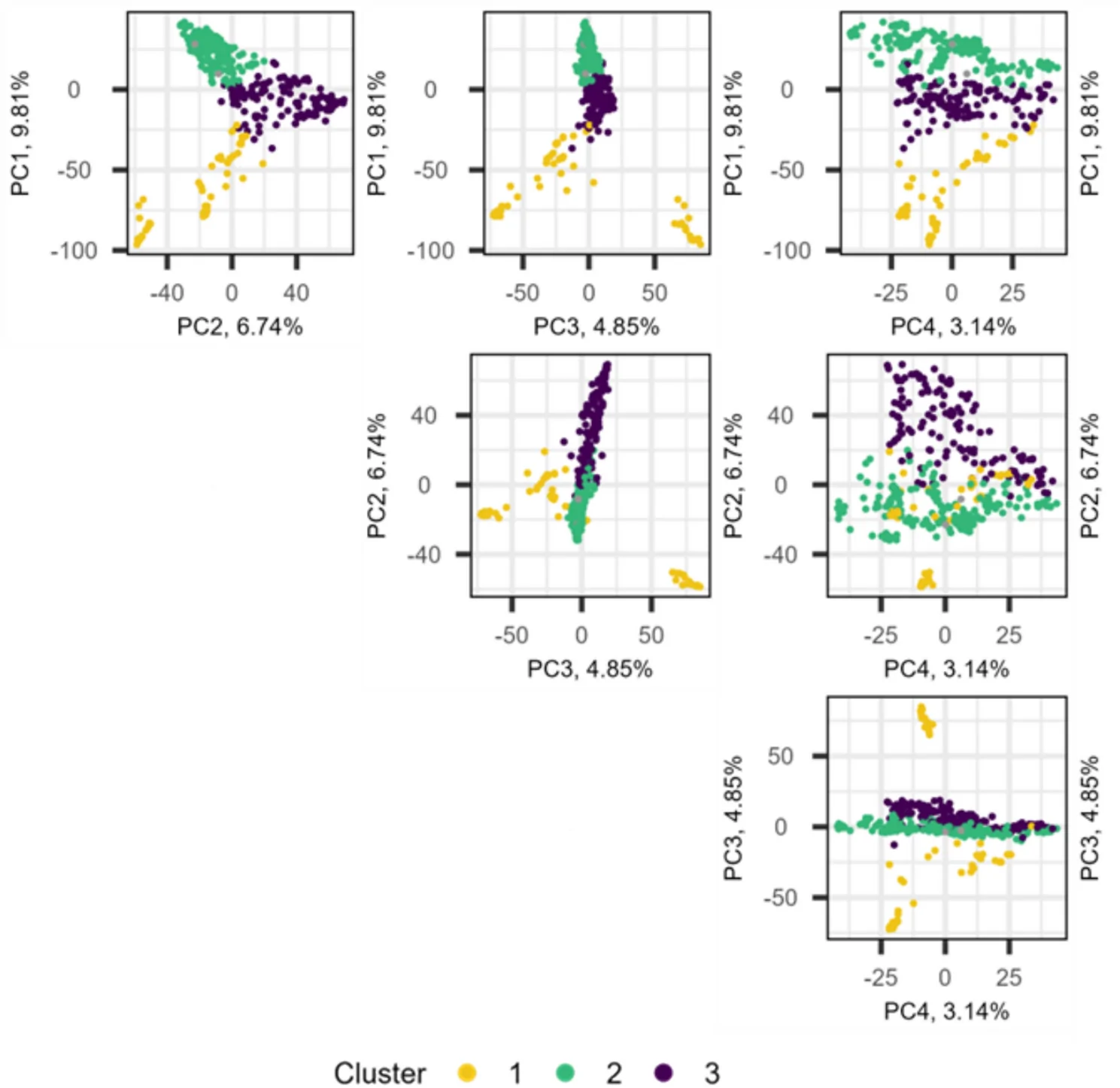
Population structure revealed by PCA of Roger’s genetic distances. Pairwise scatterplots of the first four principal components illustrate the diversity panel structure. K-means of the PCA scores identified three subpopulations (C1–C3). Axis labels indicate the proportion of total variation explained by each PC (PC1: 9.81%, PC2: 6.74%, PC3: 4.85%, PC4: 3.14%). Each point represents one genotype

Principal component analysis (PCA) indicated a moderate population structure, separating the diversity panel into three subpopulations (C1, C2, and C3; N = 135, 200, and 59, respectively) (Fig. 2). The first four principal components accounted for 9.81%, 6.74%, 4.85%, and 3.14% of the total genetic variation, respectively. These subpopulations were subsequently used to account for genetic background effects on trait expression and genotype-by-environment (G×E) interactions in downstream analyses.

Pairwise genetic correlations among environments, derived from the MET analysis, partitioned the trials into two distinct environmental clusters for each trait (Table 2). For SY, SN, and PHI, GG18 and SJR17 formed E1, whereas AS17, TEX18, TEX17, RH17, and PL17 formed cluster E2. Genetic correlations within clusters ranged from 0.32 to 0.87, with the lowest within-cluster correlation observed between GG18 and SJR17 for SY (rg = 0.32), whereas correlations between E1 and E2 were near zero or negative (− 0.31 to 0.00), indicating strong genotype re-ranking between clusters. For DTF and PH, GG18 formed E1 and AS17, plus PL17 formed E2; AS17 and PL17 were highly correlated (r_g_ = 0.91) and showed moderate correlations with GG18 (0.35–0.61). For frost survival (FS), GG21 represented E1 and JLU22 plus JLU23 constituted E2; JLU22 and JLU23 were moderately correlated (0.37), but both showed weak to negative correlations with GG21 (0.14 and − 0.49, respectively).

**Table 2:** Trait-specific environment clusters (E1, E2) defined by pairwise genetic correlations among environments (r_g_)

Trait(s)	E1	E2	Correlation within-cluster	Correlation between-cluster
SY, SN, PHI	GG18, SJR17	AS17, TEX18, TEX17, RH17, PL17	0.32–0.87	− 0.31 to 0.00
DTF, PH	GG18	AS17, PL17	0.91–0.97	0.35–0.62
FS	GG21	JLU22, JLU23	0.37	0.14 to − 0.49

PCA of environment-specific G×E BLUPs, followed by k-means clustering (k = 2), reproduced the same environment groupings as the correlation-based classification (Appendix 1 Fig. A1-A12). Subsequent analyses were therefore performed within these clusters, treating each cluster-specific phenotype as a separate trait to capture environment-specific genetic effects. Seedling vigour (SV) was not clustered and was analysed as a single trait.

### Environmental clusters and genetic subpopulations show distinct performance profiles

**Fig. 3:**
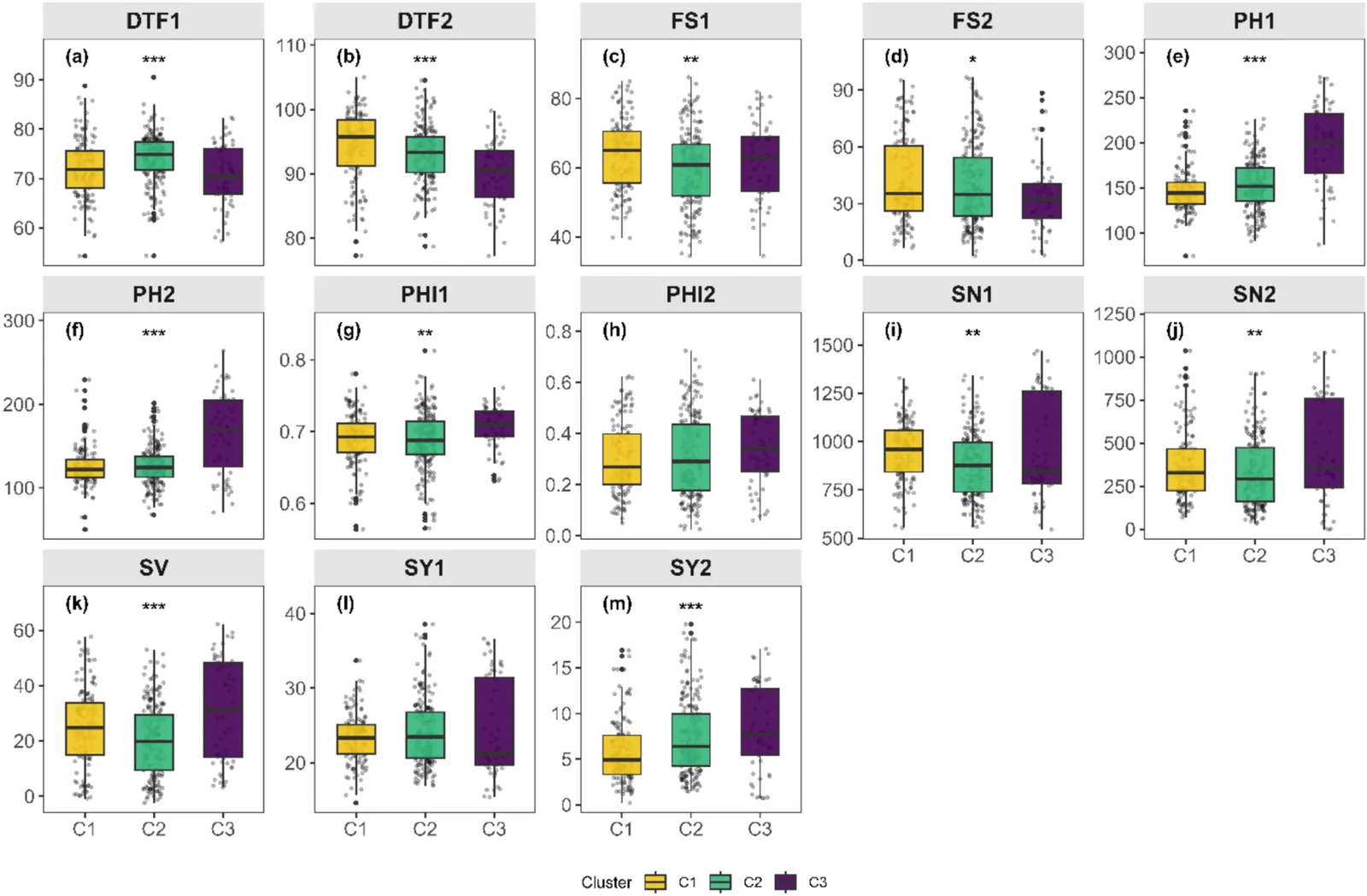
Distribution of GEBVs across subpopulations for each trait. Boxplots show the distributions of GEBVs for each trait (panels a–m) across the subpopulations (C1–C3). Trait suffixes 1 and 2 denote environment clusters E1 and E2, respectively, whereas SV was analysed as a single trait. Traits are DTF, days to flowering; FS, frost survival; PH, plant height; PHI, panicle harvest index; SN, seed number; SY, seed yield; and SV, seedling vigour. Boxes indicate the interquartile range, centre lines the median, and points individual genotypes. Asterisks indicate significant differences among subpopulations based on one-way ANOVA after FDR correction across traits (* FDR < 0.05, ** FDR < 0.01, *** FDR < 0.001)

Genomic estimated breeding values (GEBVs) revealed clear and consistent differences between environment clusters and genetic subpopulations (Fig. 3). In the full panel, E2 was associated with late DTF and lower PH, PHI, SN, and SY than E1, whereas FS was higher in E2 than E1 (Appendix 2 Table A2). Subpopulation C3 consistently exhibited higher predicted PH and a unique performance profile relative to C1 and C2 across both environmental clusters. SV was analysed separately and is reported as a single GEBV. Variance component estimates for all fitted mixed models are provided in Supplementary Table S5. To assess phenotypic differentiation among subpopulations, significant differences were tested for each trait using one-way ANOVA, and the resulting P-values were adjusted using FDR correction. Significant differences among subpopulations were detected for 11 of the 13 traits examined, whereas PHI2 and SY1 showed no significant effect.

### Environment-specific heritability varies by trait and developmental stage, with the lowest values for seedling vigour

Heritability estimates varied across traits and developmental stages (Table 3). Reproductive-stage traits exhibited moderate to high heritability in both environments, including DTF (0.79 in E1, 0.75 in E2), PH (0.71; 0.67), and SY (0.75; 0.66). Frost survival was moderate and similar across environments (0.616, 0.620). In contrast, PHI and SN showed stronger environmental dependence, with higher heritability in E1 than E2 (PHI: 0.88 vs. 0.61; SN: 0.80 vs. 0.69). Seedling vigour had the lowest heritability (0.25), indicating strong environmental influence and lower precision of genetic signal.

**Table 3:** Heritability estimates (H^2^ ± SE) for each trait within environments E1 and E2. SV was analysed as a single trait

Trait	H^2^ (E1)	SE (E1)	H^2^ (E2)	SE (E2)
DTF	0.75	0.051	0.79	0.045
PH	0.71	0.056	0.67	0.059
SY	0.75	0.053	0.66	0.056
SN	0.80	0.046	0.69	0.054
PHI	0.86	0.032	0.61	0.059
FS	0.62	0.066	0.62	0.053
SV	0.25	0.068	–	–

DTF, days to flowering; PH, plant height; SY, seed yield; SN, seed number; FS, frost survival; PHI, panicle harvest index; SV, seedling vigour; E1, environmental cluster 1; E2, environmental cluster 2

### Genetic correlation reveals stronger within-stage than between-stage correlations.

**Fig. 4:**
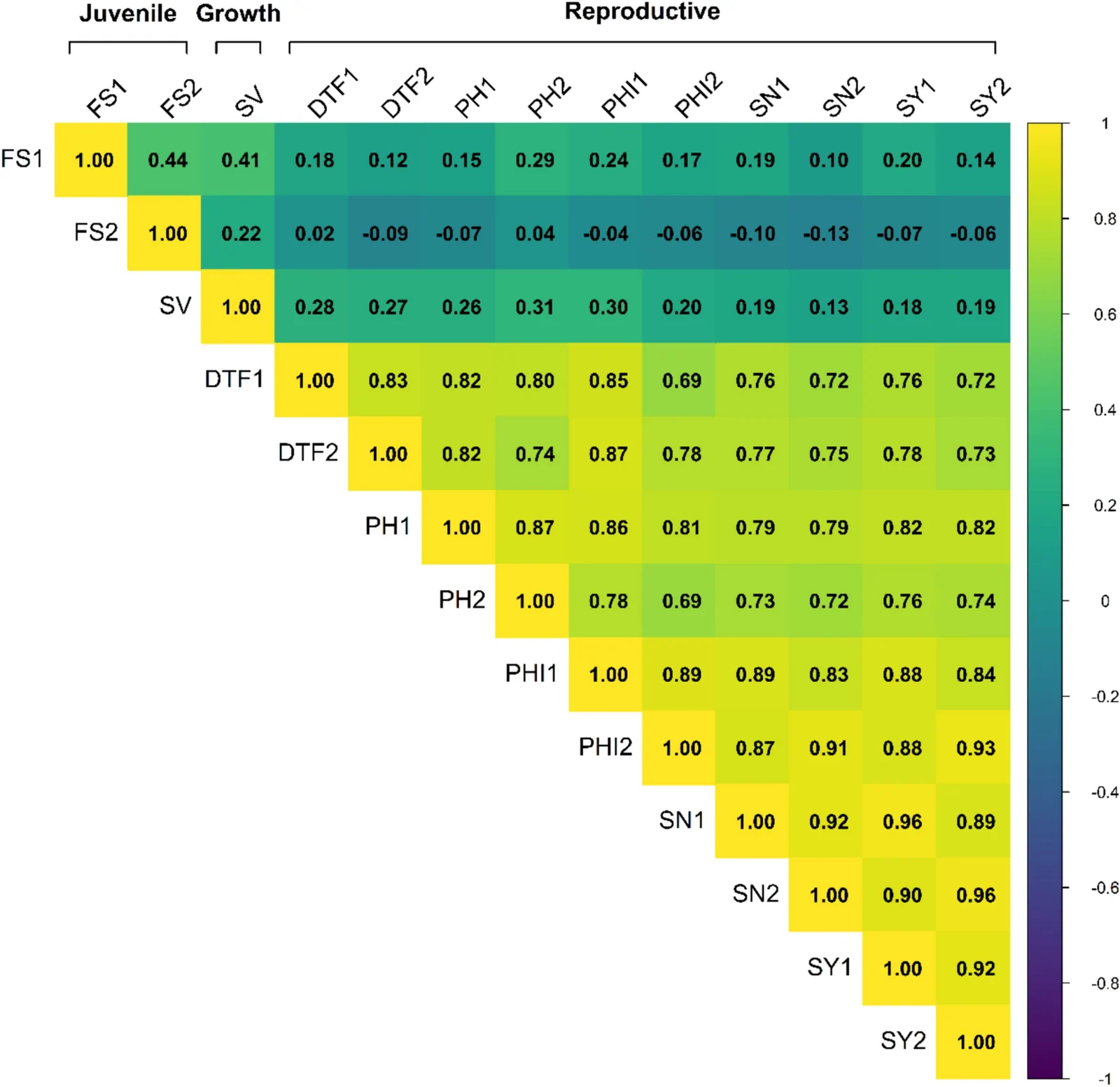
Genetic correlation matrix among traits. Pairwise genetic correlations (r_g_) estimated in MTG2 are shown as a heatmap (colour scale − 1 to 1), with values displayed in each cell. Traits are FS, frost survival; SV, seedling vigour; DTF, days to flowering; PH, plant height; PHI, panicle harvest index; SN, seed number; and SY, seed yield. Trait suffixes 1 and 2 denote environment clusters E1 and E2, respectively; SV was analysed as a single trait

Genetic correlations among traits displayed a distinct developmental stage pattern (Fig. 4). Phenology and yield traits (DTF, PH, PHI, SN, and SY) were strongly and positively correlated within environment clusters (r_g_ = 0.69 to 0.96). In contrast, early-stage traits (FS and SV) showed weak to moderate correlations with the phenology and yield trait set (r_g_ = − 0.13 to 0.41), supporting a predominantly stage-specific genetic architecture. Correlations between E1 and E2 of the same trait were high for DTF (0.83), PH (0.87), PHI (0.89), SN (0.92), and SY (0.92), while FS exhibited lower cross-environment concordance (0.44).

### Haplotype block mapping identifies variable and partly environment-specific genomic contributions

To determine the genomic distribution of genetic signals, we partitioned the genome into 380 haplotype blocks using fixed 3 cM windows, shared across all 13 traits. The number of SNPs per block varied from 2 to 98, and BlockVar showed no correlation with the number of SNPs per block (Appendix 3 Fig. A13-A25). Haplotype effects were estimated within each block using GBLUP, and variance among haplotype-specific local breeding values (BlockVar) revealed pronounced variability in genomic contributions: most blocks exhibited near-zero variances, while a limited subset showed elevated BlockVar and substantial contrasts among alternative haplotypes (Supplementary Table S3; Appendix 4 Fig. A26-A39). This pattern suggests that a limited fraction of the genome disproportionately contributes to genetic differentiation among genotypes for each trait.

To enable cross-trait comparison, blocks were ranked by BlockVar, and for each trait, we retained the smallest set of blocks accounting for 80% of cumulative BlockVar (Table 4). This threshold was chosen to capture the majority of the signal while reducing the influence of numerous low-contributing blocks. The number of blocks required to reach this threshold varied by trait, from 100 for PHI1 to 146 for DTF1 and DTF2, indicating a more polygenic architecture for the latter and a more oligogenic pattern for the former. To assess the environmental stability of haplotype contributions, we compared the 80% BlockVar sets between environments. Days to flowering (DTF) showed complete overlap between environments, with all 146 blocks shared between E1 and E2, pointing to a highly stable haplotype architecture for this trait. In contrast, FS and PHI showed pronounced environment specificity: for FS, 91 blocks were shared, with 32 unique to E1 and 36 to E2; for PHI, 78 were shared, with 22 E1-specific and 39 E2-specific blocks. These findings indicate that local genetic contributions are highly stable across environments for some traits (DTF), but highly environment-specific for others (FS and PHI), mirroring the trait-dependent GEI patterns observed in the MET analysis.

**Table 4:** Trait-specific haplotype block sets and their overlap between environments

Trait	E1	E2	Shared	E1 only	E2 only
DTF	146	146	146	0	0
FS	123	127	91	32	36
PH	111	115	87	24	28
PHI	100	117	78	22	39
SN	115	110	89	26	21
SY	105	108	87	18	21

Number of haplotype blocks required to capture 80% of cumulative BlockVar for each trait in E1 and E2, and the overlap between the corresponding block sets

DTF, days to flowering; PH, plant height; SY, seed yield; SN, seed number; PHI, panicle harvest index; SV, seedling vigour; E1, environmental cluster 1; E2, environmental cluster 2

To test whether shared blocks also had consistent haplotype effects across environments, we further assessed haplotype effect stability across all blocks (Table 5). Stability differed strongly among traits. Seed number and seed yield were the most stable, with 317 and 313 stable blocks, respectively, followed by PH with 273 stable blocks. FS and DTF were intermediate, with 228 and 183 stable blocks, respectively. PHI showed the strongest environment-specific pattern, with only 159 stable blocks and 104 environment-specific blocks.

**Table 5:** Haplotype effect stability between environments

Trait	Stable blocks	Moderately stable blocks	Environment-specific blocks
DTF	183 (48.16%)	138 (36.32%)	59 (15.53%)
FS	228 (60.00%)	105 (27.63%)	47 (12.37%)
PH	273 (71.84%)	74 (19.47%)	33 (8.68%)
PHI	159 (41.84%)	117 (30.79%)	104 (27.37%)
SN	317 (83.42%)	50 (13.16%)	13 (3.42%)
SY	313 (82.37%)	49 (12.89%)	18 (4.74%)

Values indicate the number and percentage of blocks classified as stable, moderately stable, or environment-specific. Percentages were calculated relative to the total number of haplotype blocks evaluated per trait (n = 380)

DTF, days to flowering; FS, frost survival; PH, plant height; PHI, panicle harvest index; SN, seed number; SY, seed yield

A limited number of high-variance blocks were identified as trait-specific within each environment (Table 6). For FS, 3 and 4 blocks were specific to E1 and E2, respectively. PH exhibited 7 blocks specific to E1 and 1 block specific to E2, while PHI showed 2 blocks specific to E1 and 3 specific to E2. SN in E1 contributed 2 high-variance blocks, and SV had 4. No trait-specific high-variance blocks were detected for DTF, SN in E2, or SY in either mega-environment.

**Table 6:** Trait-specific high-variance haplotype blocks and their effect ranges

Trait (unit)	Block ID	Chr	Position (cM)	Min effect	Max effect	BlockVar	# of Markers
FS1%	b000084	2	157.78	− 0.028	1.647	0.418	16
	b000246	6	107.05	− 0.145	2.041	0.299	58
	b000026	1	102.71	− 1.878	0.558	0.429	33
FS2%	b000299	8	25.76	− 0.649	1.345	0.530	15
	b000328	9	22.05	− 2.092	1.759	0.513	84
	b000302	8	36.68	− 1.273	1.549	0.633	43
	b000304	8	44.72	− 0.957	1.064	0.554	28
SV %	b000358	10	25.78	− 1.088	0.936	0.290	22
	b000217	5	116.20	− 0.553	0.546	0.236	24
	b000043	1	176.03	− 0.018	1.522	0.308	24
	b000059	2	59.35	− 1.780	0.240	0.366	35
PH1 (cm)	b000074	2	120.15	− 0.796	1.084	0.623	21
	b000350	9	118.21	− 0.397	1.975	0.752	19
	b000344	9	98.80	− 0.435	3.106	0.497	59
	b000223	6	21.58	− 0.806	1.278	0.395	44
	b000232	6	53.39	− 0.864	1.882	0.392	28
	b000277	7	67.52	-1.939	1.099	1.014	71
PH2 (cm)	b000234	6	61.49	− 0.701	0.878	0.471	6
PHI1	b000131	3	116.31	− 0.007	0.005	1.183E−05	39
	b000048	2	8.96	− 0.003	0.010	1.591E−05	19
PHI2	b000306	8	54.51	− 0.003	0.002	1.476E−06	27
	b000165	4	79.87	− 0.001	0.001	1.290E−06	17
	b000296	8	13.16	0.001	0.005	1.300E−06	40
SN1 (seed count)	b000319	8	107.13	− 15.883	0.783	17.955	34
	b000308	8	63.90	− 9.465	3.952	24.099	28

The listed blocks represent those with the largest BlockVar for a single trait, and their corresponding haplotype effect ranges. “Number of markers” is the SNP count per block

FS, frost survival; SV, seedling vigour; PH, plant height; SN, seed number; PHI, panicle harvest index; Trait suffixes 1 and 2 denote mega-environment clusters E1 and E2, respectively; SV was analysed as a single trait

### Overlap between reported SNP associations and high-BlockVar haplotype blocks

As an independent benchmark for the BlockVar approach, we further examined whether SNP associations previously reported from the same dataset by Chakrabarty et al. (2021) and Kravcov et al. (2025) were captured by haplotype blocks with high BlockVar. In total, 57 SNP associations, representing 48 unique genomic positions, were reported for SY, SN, PHI, DTF, PH, FS1, and FS2 (Supplementary Table S4). Of these, 33 of 57 associations in E1 (57.9%) and 29 of 57 associations in E2 (50.9%) were located among the top 100 blocks based on BlockVar, indicating that reported associations were frequently located within high-BlockVar blocks. This pattern was also evident among the top 50 blocks, which contained 18 of 57 associations in E1 (31.6%) and 20 of 57 associations in E2 (35.1%). For PH, four out of five significant SNP associations fell within high-BlockVar blocks; two SNPs mapped to the top-ranked block in both E1 and E2, and another SNP mapped to the second-ranked block in both mega-environments. A fourth SNP mapped to the seventh-ranked block in E1 and the 23rd-ranked block in E2 (Chakrabarty et al. 2021). For SY, one SNP mapped to the third-ranked block in E1 and the sixth-ranked block in E2. The same block also supported SN, ranking second in E1 and third in E2 (Chakrabarty et al. 2021). However, some SNPs mapped to low- to moderate-BlockVar blocks. This indicates that SNP-level significance and haplotype-level variance capture related but not identical components of the genetic signal. Nonetheless, the substantial overlap with independently reported associations supports BlockVar as a biologically meaningful metric for prioritising haplotype blocks.

### Shared high-variance blocks across traits and developmental stages suggest candidate regions with putative pleiotropic or shared effects

**Fig. 5:**
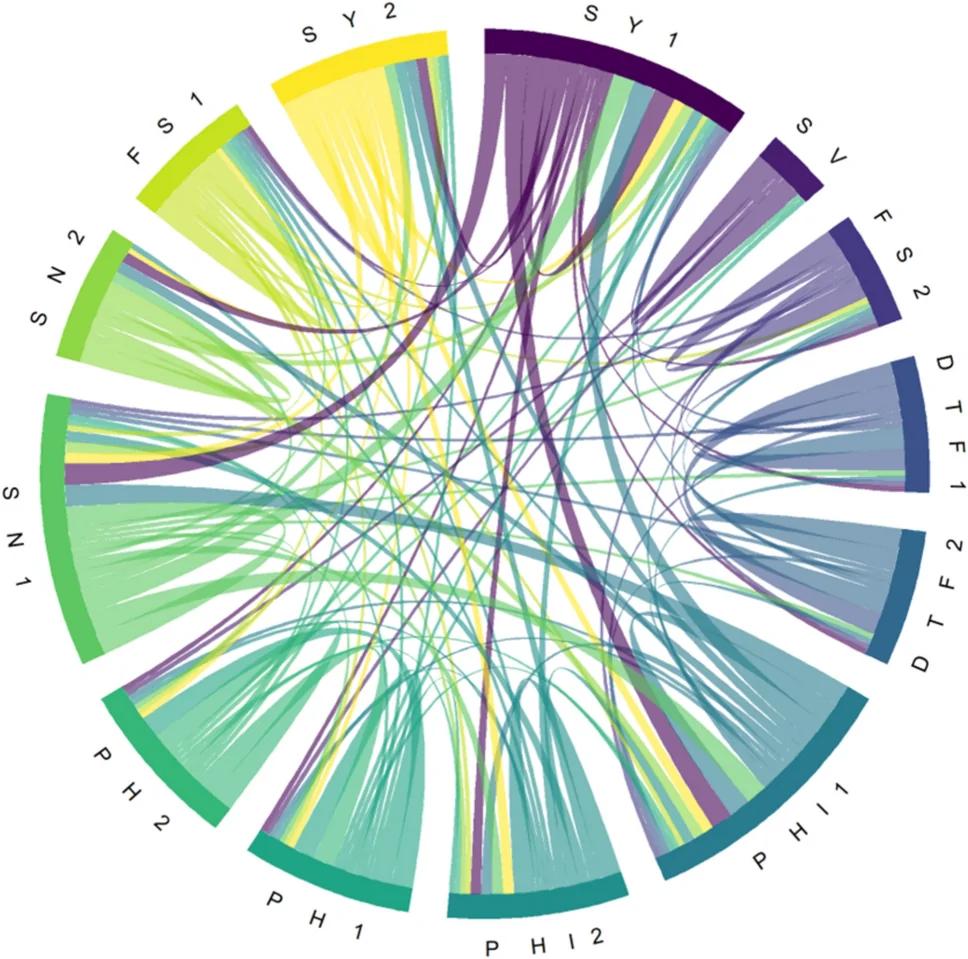
Recurrence of high-variance haplotype blocks across traits and environments. Chord diagram showing overlap among the top 10% BlockVar haplotype blocks for each trait. Each sector represents one trait; ribbons connect trait pairs that share blocks, with ribbon width proportional to the number of shared blocks. Colours denote different traits. Traits are DTF, days to flowering; FS, frost survival; PH, plant height; PHI, panicle harvest index; SN, seed number; SY, seed yield; and SV, seedling vigour. Suffixes 1 and 2 denote environment clusters E1 and E2, respectively

To determine whether multi-trait improvement could leverage shared genomic regions, we quantified the recurrence of high-variance haplotype blocks across traits (Fig. 5; Supplementary Table S2). Using the variance-based framework of Tong et al. (2024), we identified the top 10% BlockVar blocks for each trait, resulting in 128 unique blocks across the 13 traits. Of these, 98 blocks appeared in two or more traits, and 73 in three or more, indicating widespread recurrence of high-variance regions across traits and environments. Analysis across developmental stages revealed both substantial overlap and stage-specific components: Stage 1 contained 57 unique blocks, Stage 2 had 38, and Stage 3 included 110. Overlap was greatest between Stage 1 and Stage 3 (45 shared blocks), followed by Stage 2 and Stage 3 (31), and Stage 1 and Stage 2 (20); notably, 19 blocks were common to all three stages. Stage-specific blocks numbered 11 for Stage 1, 6 for Stage 2, and 53 for Stage 3. Within the reproductive stage (Stage 3), overlap patterns varied among traits, with yield-related traits sharing more blocks than phenology, suggesting a more cohesive yield module.

### In silico stacking of favourable haplotypes improves early cold tolerance and reveals trade-offs across developmental stages

**Fig. 6:**
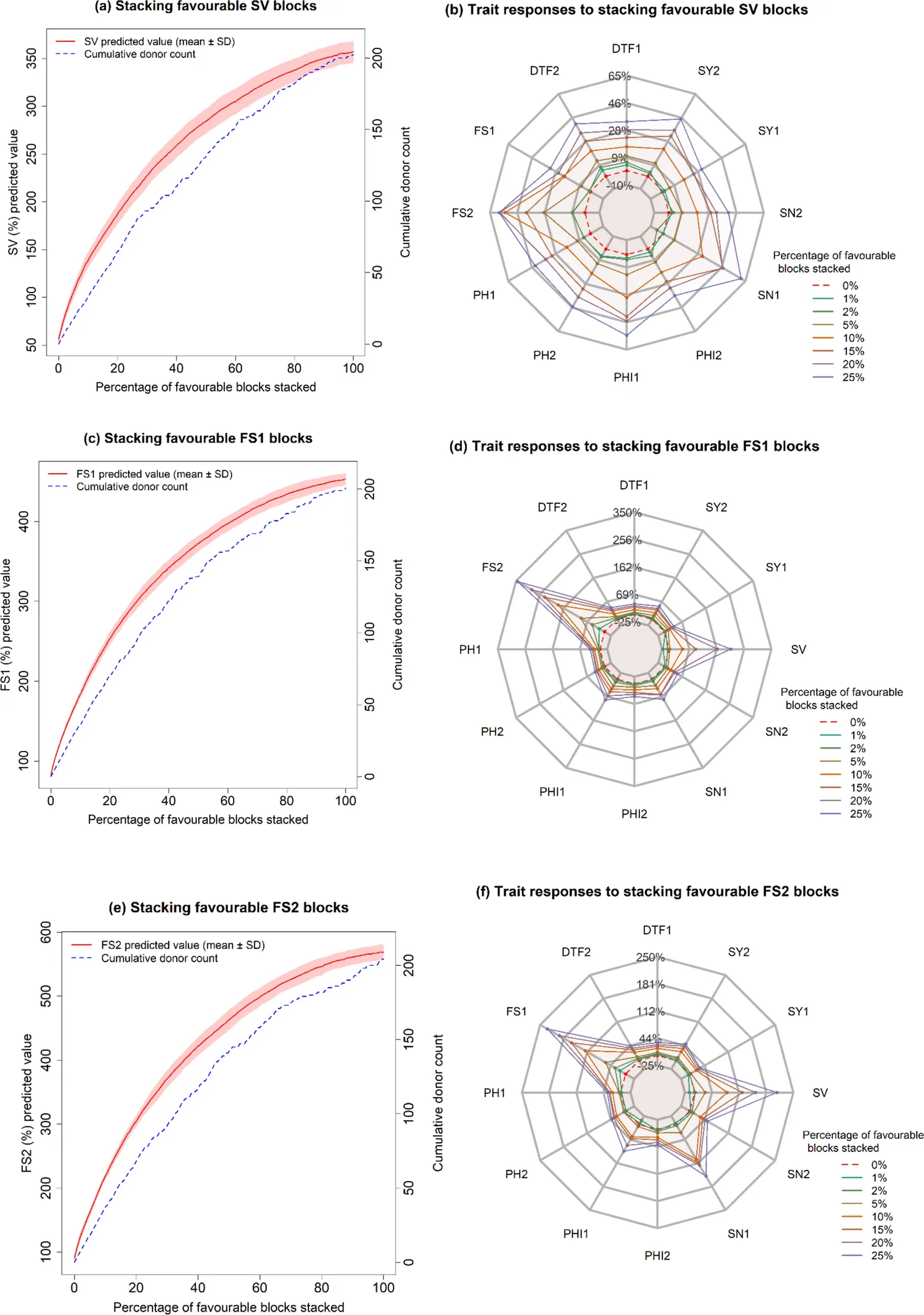
In silico haplotype block stacking for early-stage cold tolerance and correlated responses across traits. Traits are DTF, days to flowering; FS, frost survival; PH, plant height; PHI, panicle harvest index; SN, seed number; SY, seed yield; and SV, seedling vigour. Suffixes 1 and 2 denote environment clusters E1 and E2, respectively. (a,c,e) Predicted gain in SV, FS1, and FS2 as a function of stacking intensity, from 0 to 100% of top-ranked blocks stacked. Red solid lines show the mean response across nine recipient genotypes, with shaded bands indicating ± SD among recipients. Blue dashed lines indicate the cumulative number of unique donor genotypes required. Left y-axes show predicted gain relative to baseline (%), and right y-axes show donor counts. (b,d,f) Correlated responses of non-target traits to stacking for each target trait, shown as mean percentage change from baseline at discrete stacking intensities (0, 1, 2, 5, 10, 20, 50%)

Early-stage traits exhibited weak genetic correlations with reproductive traits but shared a subset of high-variance haplotype blocks. To assess the impact of stacking favourable blocks for cold tolerance, we evaluated predicted phenotype responses and correlated trait effects. Stacking progressively increased predicted performance for all three early-stage targets (FS1, FS2, and SV) as the proportion of stacked blocks rose from 0 to 100% (Fig. 6a,c,e), though gains showed diminishing returns. Among the nine recipient genotypes, mean gains at 25% stacking were +152.85% (SV), +197.77% (FS1), and +249.90% (FS2), increasing to +302%, +370.34%, and +479.34% at 100%. The number of required donors rose with stacking intensity, from approximately 73 unique donors at 25% to 158–160 at 100% (Supplementary Table S3), indicating that favourable haplotypes are broadly distributed across many genotypes.

Correlated responses in non-target traits depended strongly on stacking intensity, with unstable effects at low levels and more consistent responses at higher levels. At low stacking levels of up to 2%, some correlated responses in off-target traits were negative, but they generally increased at higher intensities (Fig. 6b, d, f; Supplementary Table S3). For FS1 stacking, SV decreased at 1–2% (− 9.54%, − 2.10%) but became positive by 5–20% stacking (+ 35.31% at 5%; +68.14% at 20%). At 20% stacking, FS1 increased by +205.64%, with the strongest responses in Stage 1 FS2 (+ 246.65%), and substantial improvements in Stage 3 traits, e.g. SY2 (+ 40.27%), PHI1 (+ 51.92%). At 50% stacking, FS1 rose by +349.27%, with even larger gains in early-stage traits (FS2 +440.03%, SV +92.88%) and Stage 3 responses ranging from +40.91 to +99.06%.

For FS2 stacking, several traits were slightly negative at 1% stacking (e.g. PHI1 − 5.03%, SY1 − 2.02%, SV − 0.46%) but shifted to positive responses by 10%, increasing further at 20%. At 20% stacking, FS2 rose by +237.86%, with strong improvements in Stage 1 (FS1 +191.02%) and Stage 2 (SV +77.15%), and smaller yet positive responses in Stage 3 (e.g. SY2 +40.46%, SN1 +46.29%). At 50% stacking, FS2 increased by +413.52%, with greater gains in early-stage traits (FS1 +326.25%, SV +129.43%) and Stage 3 responses between +40.85% and +67.22%.

When stacking favourable haplotypes for SV, the correlated responses of FS1, SN1, and SN2 were slightly negative at the 2% level (− 0.22%, − 2.22%, and − 1.16%, respectively), but became positive at higher stacking intensities. At 10%, SV increased by +150.51%, with positive responses in Stage 1 (FS2 +47.40%, FS1 +19.16%) and smaller improvements in Stage 3 (e.g. SY2 +21.00%, PHI1 +25.82%). At 50% stacking, SV increased by +412.85%, with substantial early-stage gains (FS2 +77.22%, FS1 +46.26%) and Stage 3 responses ranging from +43.14 to +65.77%.

A consistent flowering time trade-off was observed for both DTF1 and DTF2. At 10% stacking, DTF1 increased from 91.07 to 106.01 days for FS1 stacking, from 91.89 to 108.20 days for FS2 stacking, and from 92.20 to 107.30 days for SV stacking. For DTF2, the corresponding increases were from 73.50 to 88.13 days, from 71.39 to 86.86 days, and from 75.05 to 87.98 days, respectively. At 50% stacking, DTF1 reached 138.65, 136.13, and 137.44 days, while DTF2 reached 117.58, 111.87, and 114.45 days for FS1, FS2, and SV stacking, respectively. These results indicate that stacking favourable haplotypes for early-stage cold tolerance was consistently associated with delayed flowering in both environmental clusters. To assess whether this DTF-related trade-off was consistent across genetic backgrounds, stacking responses were further evaluated within C1, C2, and C3. The same general pattern observed in the full panel was retained across the three subpopulations, although the magnitude differed. At 10% stacking, the DTF delay was similar to the global response in C1 (− 1.3% to +0.8%), stronger in C2 (+ 7.0% to +8.7%), and weaker in C3 (− 26.4% to − 25.6%) across the three stacking targets.

To determine whether favourable donor haplotypes were concentrated in specific genetic backgrounds, we examined the subpopulation distribution of the strongest block effects for FS1, FS2, and SV. The strongest effects were distributed across C1, C2, and C3, rather than being restricted to one subpopulation. For FS1, top effects were observed in C1, C2, and C3 for 114, 118, and 50 blocks, respectively. The corresponding numbers were 108, 124, and 50 for FS2, and 98, 137, and 46 for SV. Many top effects were shared between two subpopulations (68 for FS1, 69 for FS2, and 75 for SV), whereas the same top effect occurred in all three subpopulations for fewer blocks (30, 29, and 24, respectively). These results indicate that favourable donor haplotypes were widely distributed across the diversity panel, helping to explain the rapid increase in donor number with increasing stacking intensity.

## Discussion

The response of sorghum to cold stress varies across developmental stages and environments, yet genetic effects are rarely quantified jointly across stages and stress scenarios. Our findings demonstrate that the genetic architecture of cold-response traits is both stage- and environment-dependent. Genetic correlations highlight opportunities for simultaneous improvement of adaptive traits for cold tolerance and end-point traits such as grain yield, but also indicate constraints imposed by trait-specific trade-offs in certain environments and developmental stage contexts. Haplotype block analyses uncovered a mosaic of trait- and stage-specific regions, as well as blocks shared among traits. Furthermore, in silico block stacking provides a quantitative framework for translating these genetic signals into expected gains and correlated responses in non-target traits.

### GxE interactions contribute to variation in cold tolerance across environments

Multi-environment analysis partitioned the testing environments into two environmental clusters (E1 and E2) that were repeatable across trait-specific analyses and corroborated by both genetic correlation clustering and PCA of environment-specific G×E BLUPs. The two clusters corresponded to clearly interpretable contrasts in environmental conditions: for reproductive-stage traits, E1 comprised the warmer environments (GG18, SJR17) while E2 included the cooler sites (AS17, TEX17, TEX18, RH17, PL17); for frost survival, the two clusters separated the open-field trial from the semi-controlled frost experiments. These contrasts support treating the clusters as distinct selection targets rather than a single target population of environments (Malosetti et al. 2013). Nevertheless, cold stress intensity likely varies continuously among environments rather than falling into discrete categories. As such environmental descriptors were not captured in the present study, future studies should integrate quantitative measures such as chilling accumulation, minimum temperature, or cold exposure during trait-specific developmental windows within reaction norm or random coefficient models to quantify how genetic effects change along cold stress gradients.

The strength of GEI varied substantially across traits. Between-cluster genetic correlations were high for DTF (r_g_ = 0.83) and PH (r_g_ = 0.87), indicating that the genetic architecture controlling phenology and plant height is largely shared between E1 and E2. This interpretation was reinforced by the haplotype block analysis, which showed complete overlap of high-variance blocks between E1 and E2 for DTF and near-complete overlap for PH. In contrast, yield-related traits (SY, SN, PHI) and FS combined lower between-cluster r_g_ values with pronounced environment specificity at the haplotype block level, consistent with substantial crossover GEI where both the genomic regions involved and their effects differ across clusters.

The practical consequences of this distinction differ by trait. For DTF and PH, selection across the two environmental clusters appears generally robust because the genetic architecture is largely shared, even though the expected gain will differ between clusters because the genetic variance is rescaled. For yield-related traits and FS, selection should be preferentially conducted within clusters, because the underlying architecture differs, and across-cluster selection risks promoting genotypes adapted to the wrong environment. The magnitude of GEI was also background-dependent across the three genetic subpopulations (C1–C3), suggesting that prediction and deployment strategies should account for genetic background as well as environmental cluster, rather than treating either dimension alone as sufficient for targeting (Buntaran et al. 2021; Fradgley et al. 2025).

This trait-dependent GEI was mirrored at the genomic level. Traits with pronounced crossover GEI retained more haplotype blocks unique to one cluster, while traits with greater stability exhibited more shared blocks between E1 and E2. However, overlap of top BlockVar regions reflects the sharing of major genomic regions, not necessarily identical haplotype effect rankings. The haplotype effect stability analysis addressed this distinction. For example, DTF showed complete overlap of high-contributing regions but only intermediate haplotype effect stability, suggesting that the same regions contributed across environments, while some within-block haplotype effects remained environment-dependent. Conversely, SN and SY showed the strongest conservation of haplotype effects, followed by PH, whereas PHI showed the strongest environment-dependent pattern. This pattern supports a mixed genomic architecture, with certain regions exerting effects primarily under specific environmental conditions and others contributing across environments. Similar mixed patterns have been reported in wheat and barley (Sehgal et al. 2020; Brunner et al. 2024; Vo Van-Zivkovic et al. 2025; Tong et al. 2025). Shared blocks are promising candidates for broad adaptation, whereas environment–specific blocks are more relevant for targeted improvement in particular environmental scenarios. However, overlap of high-importance blocks across traits or environments should be interpreted cautiously, as it may result from pleiotropy or tight linkage among loci. Additionally, the classification of blocks as “shared” or “specific” can be sensitive to the threshold used for defining high-importance blocks.

The biological relevance of the BlockVar framework was further examined by comparing top-ranked haplotype blocks with SNP associations previously reported from the same diversity panel (Chakrabarty et al. 2021; Kravcov et al. 2025). Several reported SNP associations for yield-, phenology-, plant height-, and frost survival-related traits were located within top-ranked BlockVar blocks for the corresponding traits (Supplementary Table S4), indicating that the framework recovered several genomic regions previously detected by single-marker GWAS. However, some reported SNPs were located outside the top-ranked blocks, suggesting that SNP-level significance does not always correspond to a strong block-level contribution. Thus, BlockVar provides a complementary approach for prioritising genomic regions by capturing the combined contribution of linked variants rather than simply reproducing GWAS results.

Although the uniform 3 cM window allowed consistent comparison of haplotype effects across traits, environments, and stacking scenarios, it represents an empirical approximation of LD structure. Region-specific or chromosome-specific LD-informed block definitions may capture local LD variation more precisely. This trade-off means that some haplotype blocks may span regions of locally stronger or weaker LD than the genome-wide average, so true haplotype boundaries could be split or merged relative to a locally optimised definition, and BlockVar for individual blocks should be interpreted with this in mind. Please double check it reads well in context and adjust the transition if needed.

The magnitude of the genetic signal varied among traits and environments, suggesting that the response to selection will differ across components of cold tolerance. Overall, heritability estimates were highest for reproductive traits and lowest for SV. PHI and SN also displayed environmental differences between the two environments. This stage-dependent heritability pattern has been observed previously in sorghum under cold stress, with yield traits related to reproductive cold tolerance typically exhibiting higher heritability and early-stage cold tolerance related to germination and seedling vigour traits showing lower heritability (Schaffasz et al. 2019; Vera Hernández et al. 2023). These findings reinforce the need for stage- and environment-specific genetic adaptation strategies. Accordingly, selection for reproductive-stage cold tolerance should rely on direct evaluation in the target environment, rather than assuming that early-stage traits are reliable predictors of subsequent cold tolerance (Krishnamurthy et al. 2014; Elango et al. 2022).

The lower heritability of SV does not imply the absence of genetic control, but reflects environmental sensitivity and the difficulty of measuring this trait precisely under cold stress conditions. Previous studies in sorghum indicate that growth-stage cold response is highly sensitive to environmental conditions, which can reduce the precision with which genotypic differences are estimated relative to later reproductive traits (Vera Hernández et al. 2023; Emendack et al. 2021). Nevertheless, SV remains a useful trait for growth-stage screening, although its value as a selection criterion should be validated across environments before being considered as a strong standalone predictor of later cold tolerance (Yu et al. 2003; Parra-Londono et al. 2017; Emendack et al. 2021). At the same time, since SV was scored at fixed days after sowing rather than at matched developmental stages, developmental asynchrony among genotypes likely inflated residual variance, contributing to the low heritability estimate.

Cold tolerance exhibited a stage-dependent genetic architecture. Weak genetic correlations across stages indicate that selection at one stage will only partially translate to performance at other stages. This is consistent with prior QTL and GWAS studies in sorghum, which identified that early- and reproductive-stage cold tolerance are largely non-overlapping genomic regions underlying early- and reproductive-stage cold tolerance (Chakrabarty et al. 2021; Neitzert et al. 2025; Parra-Londono et al., Parra-Londono et al. 2017; Elango et al. 2022). Our haplotype block analysis reinforces this pattern: many high-variance blocks were specific to individual stages, while others were shared across traits from different stages, reflecting both a common genomic component and stage-specific effects. Shared high-variance regions may reflect putative pleiotropy or tight linkage among loci affecting traits at different stages (Chebib and Guillaume 2021). The existence of trait- and stage-specific blocks indicates that a substantial fraction of genetic variance is limited to certain developmental stages, which is valuable for breeding stage-specific targets such as reproductive performance. Analysis of top-decile BlockVar regions supports this, revealing asymmetric overlap across developmental stages, with Stage 3 exhibiting the largest stage-specific component (Fig. 5). These findings suggest that reproductive-stage cold response relies on both a shared genomic structure and additional stage-specific regions. Field studies have repeatedly shown that seedling cold tolerance scores are informative for initial screening but are weak predictors of overall cold stress performance in sorghum (Maulana and Tesso 2013; Ostmeyer et al., 2020; Neitzert et al. 2025). Our haplotype block results provide a genomic explanation, showing that much of the variance underlying reproductive-stage response resides in blocks distinct from those active at earlier stages. Selection programmes targeting reproductive resilience will therefore require dedicated phenotyping at that stage rather than extrapolation from seedling assays.

### Haplotype stacking enables in silico assessment of potential genetic gain and trade-offs

Our haplotype block findings support a mixed genetic architecture for cold tolerance, with some blocks shared across traits and others exhibiting trait specificity. This is directly relevant to how breeding objectives are defined: broadly acting haplotypes can be deployed to raise baseline performance across multiple stages or environments, while context-dependent haplotypes are better suited to objectives targeting specific stress scenarios or developmental windows, such as reproductive-stage cold resilience in temperate production zones.

For FS1, FS2, and SV, predicted gains rose rapidly at low to moderate stacking levels, with a 100% gain achieved by stacking only 5.26% of blocks for FS2 (20 blocks), 6.58% for FS1 (25 blocks), and 10.26% for SV (39 blocks), indicating an oligogenic-to-moderately polygenic architecture for these traits. This pattern suggests that initial gains are concentrated in a limited subset of high-impact blocks, with additional blocks contributing more gradually as stacking proceeds. Similar in silico haplotype stacking approaches in wheat have shown that a substantial proportion of the achievable gain was captured by stacking a limited subset of high-impact haploblocks, with progressively smaller increments as additional blocks are added (Tong et al. 2024); these findings sit within a broader framework in which haplotype-based selection can preserve useful diversity while delivering long-term gain (Villiers et al. 2024). As expected, as stacking intensity increased, the number of required donors rose rapidly, which indicates that favourable blocks are spread across many genotypes rather than being concentrated in a few elite parents. This pattern is consistent with the diversity panel structure of our population, while in a narrower elite line set, favourable haplotypes would likely be concentrated in fewer donors. This shifts the breeding challenge from identifying useful haplotypes to efficiently combining them in realistic crossing schemes. In this respect, our results are consistent with genomic mating and optimal cross-selection studies, which show that once favourable alleles are dispersed across multiple lines, the key challenge is to design crosses that combine them efficiently while maintaining diversity (Akdemir and Sánchez 2016; Gorjanc et al. 2018; Allier et al. 2019; Ahadi et al. 2024). However, the predicted trait values exceeding 100% in these simulations should be interpreted as a modelling artefact: our model sums favourable haplotype effects without imposing an upper bound, whereas the trait itself is biologically constrained to 0–100%. In addition, the stacking procedure combines haplotypes from genetic backgrounds that do not co-occur, which inflates predictions further. For this reason, the stacking results should be interpreted primarily in terms of relative patterns, including the efficiency of gain accumulation, diminishing returns with increasing stacking intensity, and the associated donor and crossing complexity, rather than as direct predictions of biologically attainable gain magnitudes. Moreover, the in silico stacking approach assumed additive accumulation of haplotype block effects and did not explicitly model epistatic or other genetic background-dependent interactions among blocks. In real breeding populations, non-additive effects may also contribute to saturation of predicted gains. Taken together, these results reinforce that early-stage cold tolerance improvement will rely on combining haplotypes across, rather than within subpopulations.

The correlated responses to haplotype stacking show that early-stage cold tolerance cannot be improved independently of other traits. Blocks were stacked in descending order of BlockVar for the target trait, so the first blocks added were those with the largest individual contributions, and subsequent blocks contributed progressively less. Correlated responses varied with stacking intensity: at very low stacking levels, responses were unstable or even changed direction, but at moderate levels they became more consistent. This suggests that the first few stacked blocks reflect trait-specific blocks, while stacking larger sets increasingly reflects the underlying genetic covariance structure among traits, which is expected as the portion of the genome under selection expands with increased percentages of selected genome-wide haplotype blocks. This implies that block selection should account not only for the target trait, but also for correlated effects on other traits (Wartha and Lorenz 2024; Cerón-Rojas et al. 2026). The pattern was also stage-dependent: stacking for FS1, FS2, and SV produced stronger correlated responses among early-stage traits than among reproductive traits, indicating that genetic sharing is stronger within developmental stages than across stages. This pattern is consistent with evidence showing that sorghum cold tolerance mechanisms vary across growth stages (H. Yang et al. 2025; Neitzert et al. 2026), and also with simulation-based analyses showing that improving a target trait through haplotype stacking can generate correlated changes in non-target traits depending on the genetic correlation structure (Tong et al. 2025).

In silico stacking of haplotypes for improved early-stage cold tolerance tended to increase DTF1 and DTF2, resulting in a predicted delay in flowering. This is consistent with field observations that early-season cold stress delays flowering and maturity in sorghum (Ostmeyer et al. 2020), and suggests partial genetic overlap or tight linkage between regions controlling early cold response and those influencing phenology. Although the magnitude of this shift was generally modest at lower stacking levels, the result highlights a potential trade-off, as such delays are undesirable when adaptation requires flowering within a specific production window. Therefore, improvement of early-stage cold tolerance should be evaluated jointly with phenology, for example, by excluding blocks with unfavourable effects on DTF or by applying multi-trait selection weights, rather than focusing on the target trait alone (Moeinizade et al. 2020). The subpopulation-specific stacking analysis further indicates that the trade-off pattern was broadly conserved across genetic backgrounds, although the magnitude of the response differed among C1, C2, and C3. Accordingly, the stacking exercise is most useful as an in silico indicator of potential gain and trade-off with a given donor set, rather than serving as a predictor of breeding outcomes.

Together, these results outline an integrated strategy for improving cold tolerance in sorghum that separates broadly transferable targets from environment-specific targets. The two repeatable environmental clusters define where selection should be conducted separately rather than pooled, and the trait-dependent strength of GEI tells which traits demand environmental-specific evaluation and which can be selected with broader confidence. Stable haplotype effects, especially for SN, SY, and PH, are more suitable for broad adaptation strategies, whereas environment-specific effects, particularly for PHI, FS, and partly DTF, support targeted selection within specific environmental clusters. The stage-partitioned haplotype architecture, with a small recurring core across stages, reframes early- and reproductive-stage cold tolerance as a structured multi-target breeding problem rather than two independent breeding objectives: the shared blocks support a baseline gain at both stages, while the larger stage-specific component requires dedicated phenotyping at the reproductive stage and cannot be inferred from seedling assays. The in silico stacking analysis adds a quantitative layer to this picture, showing that most of the achievable gain in early-stage tolerance is concentrated in a small subset of high-impact blocks, that favourable haplotypes are dispersed across many donors rather than concentrated in a few elite lines, and that improvement of early-stage tolerance carries a consistent delay in flowering, a trade-off that would otherwise emerge only after years of late-stage field testing. The framework is necessarily an in silico indicator and not a substitute for empirical validation, but it converts haplotype-level genetic signals into the kind of trade-off information that breeding decisions actually require.

## Conclusion

Sorghum cold tolerance has so far mostly been treated as either a single breeding target evaluated at one stage, or as a set of separate challenges handled independently at germination, seedling, and reproductive stages. Neither adequately reflects the genetic architecture we describe here. Cold tolerance in sorghum is neither a single, unified target nor a set of fully independent stage-specific traits; rather, it emerges from a structured combination of stage-specific and shared genomic components, with predictable trade-offs between them. The practical consequence is that breeding gain for cold tolerance in temperate sorghum is no longer constrained primarily by a lack of phenotypic information or genomic resolution, but by the need to design selection schemes that can integrate information across developmental stages while accounting for the correlated response in phenology. Developing and validating such schemes is therefore a key next step for translating these results into cultivars adapted to cool season production environments where cold tolerance currently limits sorghum cultivation.

## Supplementary Information

Below is the link to the electronic supplementary material.

Supplementary file1 (PPTX 1473 KB)

Supplementary file2 (XLSX 571 KB)

Supplementary file3 (XLSX 1253 KB)

Supplementary file4 (CSV 8 KB)

Supplementary file5 (XLSX 19 KB)

Supplementary file6 (XLSX 11 KB)

Supplementary file7 (DOCX 1727 KB)

Supplementary file8 (DOCX 1721 KB)

## Data Availability

The data analysed in this study include phenotypic, genotypic, and environmental information from published and partner-generated datasets. Publicly available datasets are cited in the manuscript (Chakrabarty et al. 2021; Kravcov et al. 2025). Additional processed data, model outputs, and analysis scripts are available from the corresponding author upon reasonable request and subject to institutional and partner data-sharing agreements.
