# Supplementary material for: Genetic dissection of cold tolerance across developmental stages in sorghum reveals stage-specific haplotypes and potential trade-offs
Source: Theor Appl Genet. 2026 Sep 4;139(9):258. doi: 10.1007/s00122-026-05356-w (PMC13545123; doi:10.1007/s00122-026-05356-w)
Supplement: Supplementary file 1 [file 122_2026_5356_MOESM7_ESM.docx]

**Appendix 3: Genome-wide distribution of block-specific variance (BlockVar) across traits.**

**figure A13 and A14: SN1 & SN2**

**figure A15 and A16: SY1 & SY2**

**figure A17 and A18: PHI1 & PHI2**

**figure A19 and A20: DTF1 & DTF2**

**figure A21 and A22: PH1 & PH2**

**figure A23 and A24: FS1 & FS2**

**figure A25: SV**

Fig (A13 to A25). Block-specific variance (BlockVar) across 380 fixed 3 cM haplotype blocks for 13 traits. Figures A13–A24 represent raits DTF, days to flowering; FS, frost survival; PH, plant height; PHI, panicle harvest index; SN, seed number; SY, seed yield; and SV, seedling vigour. Trait suffixes 1 and 2 denote environment clusters E1 and E2, respectively; SV was analysed as a single trait..
